# Editorial: CRISPR tools, technology development, and application

**DOI:** 10.3389/fpls.2023.1329780

**Published:** 2023-11-13

**Authors:** Penna Suprasanna, Magdalena Klimek-Chodacka, Shri Mohan Jain

**Affiliations:** ^1^ Amity Centre for Nuclear Biotechnology, Amity Institute of Biotechnology, Amity University of Maharashtra, Mumbai, India; ^2^ Faculty of Biotechnology and Horticulture, University of Agriculture in Krakow, Krakow, Poland; ^3^ Department of Plant Production, University of Helsinki, Helsinki, Finland

**Keywords:** CRISPR-Cas, genome editing, crop plants, anti CRISPR proteins, transformation, epigenome, rice, virus-induced genome editing

CRISPR/Cas-based genome editing tools have revolutionized nearly every field of life sciences, especially the plant biology (Hu and Li, 2022). The techniques have added a new dimension to basic research to study the genes’ function through their knockout or activation. The main significant application of the CRISPR system has been to develop targeted genetic modification in plants to cope better in a changing climate that is becoming less favorable to achieve higher plant productivity. The use of precise genome editing has been shown to be much safer than traditional mutagenesis or transgenics, especially since the changes often involve single nucleotides and are not necessarily related to the presence of foreign DNA in the modified genome (El-Mounadi et al., 2020; Jung and Till, 2021). Even though CRISPR tools are very dynamically developed and constantly improved, there are still many challenges that must be overcome. In this Research Topic, we made attempts to showcase the prospects for efficient and precise editing of plant genomes, as well as present their application to overcome current issues in plant biology and food security. Currently, many tools have been developed to allow editing of the target loci. Unfortunately, the tools that are often available show low efficiency for certain plant species or tend to induce unintended mutations at off-target sites. The possibility of achieving efficient genome editing is also directly based on the development of transformation techniques and the delivery of essential CRISPR system components to plant cells, which is often much more complicated than in the case of animal cells.

In case of several horticultural crops, transgenic breeding has led to creation of genetically modified plants (Ghag et al., 2022), however genome editing has been successfully achieved in some vegetables. The transgenic plant development in broccoli has majorly focused on nutritional quality and stress resistance. One of the important diseases occurring worldwide, is the clubroot disease caused by *Plasmodiophora brassicae* affecting rapeseed, cauliflower, broccoli, Brussels sprouts, Chinese cabbage, and radish. Hence there is a need to develop protocols for targeted manipulation of the resistance genes into cultivars. Zhao et al. established an efficient transformation system based on *Agrobacterium* sp. which can be useful for transgenic and genomic editing. The authors tested different broccoli genotypes, transformation vectors, RNAi and CRISPR/Cas9, and selection agents. The results of the study provide a platform for further studies on the development of transgenic and gene editing technologies in broccoli and other cruciferous crops.

Among the important vegetables, tomato stands as an economically important, functional food due to several health promoting bioactive metabolites such as lycopene, beta-carotene, vitamins etc. (Ali et al., 2021). This crop also has been a good candidate for genome editing studies and there have been several reports on genome editing and characterization of plants for a wide range of traits including plant architecture and flower characters, fruit ripening, nutritional quality, and biotic and abiotic stress tolerance (Egea et al., 2022). Tiwari et al. presented a detailed account of the advances in CRISPR/Cas based technologies for use in tomato improvement. Authors have also outlined the design of the guide RNA and CRISPR/Cas9 constructs, transformation protocols, diversity in CRISPR/Cas toolbox, and base/prime editing. This mini-review also highlighted different applications of genome editing of different traits useful in breeding of highly productive tomato crop.

Verma et al. reviewed the latest advancements in plant genome editing technologies (base editing, prime editing, multiplex gene editing, epigenome editing, gene delivery methods) while detailing the potential roles that this technology has in store for the identification of novel gene functions to improve the traits of commercial importance. The authors have well described the applications of gene editing for the improvement of consumer-demanded traits such as higher nutritional value, colour, texture, aroma/flavour, and production of industrial products such as biofuel, fibre, rubber and pharmaceuticals. Epigenetic alterations can result in the creation of new epialleles which may have relevance for the improvement of agronomic traits like yield, nutritional quality, and stress tolerance (Fang et al., 2023). For example, the histone variant Sl_H2A.Z has a functional role in the development and ripening of tomato fruits (Yang et al., 2021), and histone modifications regulate flowering efficiency and grain size that determine yield in rice (Shi et al., 2015). While mentioning that the genome editing technology has certain challenges like off-targeting, organellar genome editing and ploidy changes, the authors stressed the need for further research and universal regulatory framework on genome editing crops.

Anti-CRISPR proteins, discovered in phages (Bondy-Denomy et al., 2013), have been exploited to prevent CRISPR/Cas-mediated gene editing and gene activation in plants via the regulation of off-targeted mutations and inhibiting Cas protein–editing operations (Yang et al., 2023). In the article, Choudhary et al. have outlined different mechanisms of inhibiting CRISPR-Cas9–based genome-editing tools, and how engineering of plant genomes for trait improvement can be efficiently managed through regulatory mechanisms (Calvache et al., 2022). Acr proteins like AcrIIA4 and AcrIIA5 inhibiting SpCas9, and all Cas9 orthologs respectively, have been evaluated in both herbaceous and woody plant species (Liu et al., 2023). The field of Acr proteins has now opened up newer opportunities in crop improvement for cell type-specific genome editing and inducible plant genome editing (Bubeck et al., 2018; Hoffmann et al., 2019). The authors also surveyed the literature suggesting that Acr proteins could be used to modify the insertion, deletion, silence, and single-letter fixation of any functional trait.

Most of the genome editing reports in barley are based on *Agrobacetrium* mediated transformation but the system has shown to be highly genotype dependant with some experimental limitations (Han et al., 2020). This necessitates the development of rapid, genotype independent transformation methods. The advent of virus-induced genome editing has become very useful in different plants (Uranga and Daròs, 2022). In their work, Tamilselvan-Nattar-Amutha et al. showed testing of the barley stripe mosaic virus (BSMV) in Cas9-transgenic barley plants for editing of the ALBOSTRIANS gene (CMF7) that led to albino/variegated chloroplast-defective mutants. In addition, the authors also achieved successful editing of the meiosis-related candidate genes ASY1 encoding axis-localized HORMA domain protein, MUS81 encoding a DNA structure-selective endonuclease, and ZYP1 encoding a transverse filament protein of the synaptonemal complex) in barley. The BSMV mediated VIGE gene editing has also shown applicability in wheat (Chen et al., 2022a) and cotton (Chen et al., 2022b) suggesting that BSMV with its broad host range can find significant application in crop plants.

Rice is the most important staple food crop, however rice production if often threatened by climate change, and incidence of abiotic and biotic stresses including insects and pests (Mishra et al., 2021). Among the diseases, bacterial leaf streak (BLS) and rice blast cause significant yield losses, and hence breeding strategies have been employed to develop resistant cultivars but the development of broad spectrum resistance in case of blast disease and selection of BLS resistance being a quantitative trait, are often the demanding challenges. Yang et al. developed disease resistant mutants in a susceptible material 58B by the CRISPR/Cas9 based editing of target of the Pi21 gene and a target of the effector-binding element (EBE) of the OsSULTR3;6 gene. The resultant mutant plants showed upregulation of defense responsive genes and lessened area of lesion characteristic of rice blast and bacterial leaf streak. The study can be seen as a significant step in the generation of rice varieties resistant to rice blast and bacterial leaf streak, for use in breeding and improvement of rice varieties.

Genome editing in horticultural crops has progressed with rapid genomics advancements. Despite availability of onion genome sequence information in the past few years (Finkers et al., 2021), genome editing research in onion has not progressed much. This warrants studies on the optimization of different conditions for efficient gene editing method, and evaluation of the strategy for use in onion improvement. In a first report, Mainkar et al. presented their results on the establishment of genome editing system based on a CRISPR/Cas9 system for targeting the gene encoding for Phytoene desaturase (PDS) of the Indian short-day onion. The PDS gene involved in carotenoid biosynthesis pathway has enabled researchers to identify the gene-edited phenotype based on the albino phenotype. The authors identified the *AcPDS* gene used it to evaluate the CRISPR/Cas9 based editing efficiency. The AcPDS knockout exhibited a visible mutant phenotype within a short span of 8-weeks, and the muations were mostly of InDels and substitutions type. The study can be useful for further studies as a valuable method to achieve high efficiency genome editing for different commercial traits in onion.

Among the legumes, common bean (*Phaseolus vulgaris* L.) provides substantial dietary protein and essential nutrients. Genetic engineering attempts in common bean are limited by experimental factors such as prolonged process, low efficacy, and genotype dependence, and thus there is a need for a rapid and efficient screening method to validate sgRNA efficiency. de Koning et al. described a rapid method of hairy root transformation for assessing sgRNA efficiency in common bean. *Rhizobium rhizogenes* mediated hairy root induction, has been useful as a ideal system for studying gene function in plants (Gutierrez-Valdes et al., 2020). The authors tested three different methods of hairy root induction for use in common bean cv. CIAP7247F, and employed the method to assess the *in planta* efficiency of in silico-designed sgRNAs targeting genes from the raffinose family oligosaccharides (RFOs) metabolic pathway in common bean. The study highlighted the use of hairy root transformation system for the speedy assessment of multiple sgRNAs and promoters.

## Author contributions

SP: Conceptualization, Writing – original draft, Writing – review & editing. MK-C: Writing – review & editing. SJ: Writing – review & editing.
